# Qualitative evaluation of postdoctoral trainee and faculty advisor experiences within a research-intensive school of pharmacy

**DOI:** 10.1186/s12909-022-03750-8

**Published:** 2022-09-24

**Authors:** JE McLaughlin, KA Morbitzer, F Hahn, L Minshew, KLR Brouwer

**Affiliations:** 1 Division of Practice Advancement and Clinical Education, Center for Innovative Pharmacy Education and Research, UNC Eshelman School of Pharmacy, University of North Carolina at Chapel Hill, Chapel Hill, NC 27599 USA; 2 Department of Clinical and Administrative Sciences, Xavier University of Louisiana College of Pharmacy, New Orleans, LA USA; 3 Department of Clinical Sciences, MCW Pharmacy School, Human-Centered Design Lab, Robert and Patricia Kern Institute for the Transformation of Medical Education, Medical College of Wisconsin, Milwaukee, WI USA; 4 Division of Pharmacotherapy and Experimental Therapeutics, UNC Eshelman School of Pharmacy, UNC Chapel Hill, Chapel Hill, NC USA

**Keywords:** Postdoctoral, Program evaluation, Qualitative, Pharmacy education, Research

## Abstract

**Background:**

Postdoctoral trainees play a vital role in securing grant funding, building alliances, and mentoring graduate students under the guidance of a mentor who can help develop their intellectual independence. However, the experiences of postdoctoral trainees, particularly within health professions schools, is largely unexplored. The purpose of this study was to investigate the experiences of postdoctoral trainees and faculty advisors at a public four-year school of pharmacy and identify areas of opportunity to improve postdoctoral training.

**Methods:**

Focus groups and interviews were conducted to elicit participants’ experiences, perceptions, and suggestions for improvement. Stakeholder groups included postdoctoral trainees and faculty who serve as postdoctoral advisors. Thematic coding was used to identify semantic themes, and summaries of participant perceptions were generated. Results were mapped to the identity-trajectory framework.

**Results:**

Participants described various experiences related to intellectual growth, networking opportunities, and institutional support. In addition, participant agency was critical for developing career goals and navigating transitions. COVID-19 introduced unique challenges associated with transitioning to remote work and managing goals/motivation. Areas of opportunity were identified, such as improving infrastructure, enhancing mentoring, and enhancing communication.

**Conclusion:**

Postdoctoral trainees play a critical role in the success of academic institutions. Scholarly endeavors that explore postdoctoral experiences, specifically those utilizing qualitative methods, can help pharmacy education better understand and meet the needs of postdoctoral trainees and faculty advisors. This study provides insight into the experiences of postdoctoral scholars and provides evidence for improving these training programs in schools of pharmacy.

**Supplementary Information:**

The online version contains supplementary material available at 10.1186/s12909-022-03750-8.

## Background

Postdoctoral trainees, colloquially known as “postdocs,” play a vital role in research and development at academic institutions [1]. Under the guidance of a mentor who can help develop their intellectual independence, postdocs elevate the academic strength of institutions by conducting research, assisting in securing grant funding, building alliances, and mentoring graduate students [1, 2]. In return, institutions and faculty should support their postdocs with appropriate working conditions, compensation, recognition, and career development services [2].

By definition, postdocs hold doctoral degrees (e.g., Doctor of Philosophy, Doctor of Pharmacy) and typically engage in activities that promote scholarly autonomy, disciplinary specialization, and in some cases, entrepreneurial skills [1, 3]. Their development is a complex, multifaceted process that is influenced by various factors, including mentoring, culture, and resources [1, 4–10]. Having a positive and nurturing research environment, support for career independence, and a sense of professional identity, for example, can positively impact postdoc productivity and career placement [5, 7, 10]. Unfortunately, research on postdoc development is sparse, leaving an incomplete understanding of how postdocs experience and navigate their training [1].

The identity-trajectory framework posits the role of individual agency (i.e., one's independent capability or ability to act on one's will) in academic work and career decisions [6]. Chen and colleagues described three strands of postdoc identity-trajectory (*intellectual, networking*, *institutional*), in addition to agency [6, 11]. The intellectual strand includes activities that advance one’s disciplinary specialism, such as writing, coursework, and conducting research. The networking strand embodies efforts toward building work relationships and connections to the larger scholarly community, such as membership in disciplinary organizations, collaborative research, and reviewing manuscripts. The institutional strand represents responsibilities associated with an institutional appointment (e.g., committee service, teaching) and accessing resources, such as laboratory space, funding, and supervision. Notably, agency represents the postdocs’ motivations, intentions, personal aspirations, and work goals while recognizing the influence of structural and systematic factors beyond their control (e.g., organizational hierarchies, government regulations, expectations of others) [6, 11]. Taken together, these elements represent the ongoing learning and development that individuals experience as postdocs, with consideration to the structures that can support and constrain their agency [6].

Although scholars from various disciplines have explored postdoc development and success [1, 6, 11–13], there is a dearth of literature regarding postdoc experiences within the health professions and specifically within pharmacy and pharmaceutical education [14–16]. This is somewhat surprising given pharmacy’s long-standing interest and investment in postdoc positions [16, 17]. At this time, research is needed to better understand the experiences of these trainees [18]. The purpose of this evaluation was to gain insight into the experience of postdocs and faculty advisors at the UNC Eshelman School of Pharmacy. The findings were mapped to the identity-trajectory framework [6, 11] and utilized to identify recommendations for supporting postdocs within schools of pharmacy.

## Methods

The UNC Eshelman School of Pharmacy has a rich history of postdoc training, with approximately 100 trainees and 60 faculty advisors across the School’s five divisions: Chemical Biology and Medicinal Chemistry; Pharmacoengineering and Molecular Pharmaceutics; Pharmacotherapy and Experimental Therapeutics; Practice Advancement and Clinical Education; and Pharmaceutical Outcomes and Policy. Some are part of the School’s multiple National Institutes of Health (NIH)-funded T32 training programs, such as the NIGMS-funded UNC-Duke Collaborative Clinical Pharmacology Postdoctoral Training Program, some are funded and jointly supervised through industry partnerships, and others are supported by other funds (e.g., grants, contracts or awards to principal investigators) and not associated with a cohort-based training program. Further, degree qualifications for postdocs can vary, including Doctor of Pharmacy (PharmD), Doctor of Philosophy (PhD), and Doctor of Medicine (MD). PhD postdocs come from numerous fields, including chemistry, engineering, public health, library sciences, and education. Prior training (before, during, or after the doctoral degree) can also vary significantly.

Focus groups and interviews were conducted between March and December 2020 to provide an in-depth view of experiences within postdoc training. Purposive convenience sampling was used and participants were recruited via email. Any postdoc currently employed at the school and any faculty member currently advising a postdoc was considered eligible to participate. Faculty advisors were included to provide a more comprehensive view (e.g., additional perspectives) of postdoc experiences. Seven one-hour focus groups were conducted with current postdoctoral trainees (*n* = 18 in five groups) and faculty advisors (*n* = 8 in two groups). To accommodate scheduling constraints, individual one-hour interviews also were conducted with two trainees (*n* = 20 total postdocs). Scripts were structured to elicit participants’ experiences, perceptions, and suggestions for opportunity regarding postdoc training (Additional file 1: Appendix 1). For example, postdocs were prompted, “What have you gained from your postdoc training to date?” whereas faculty advisors were asked, “What strategies do you currently use to support your postdoctoral fellows?” The questions were intentionally broad to elicit participants’ overall perceptions of and experiences with postdoc training regardless of duration. Probing questions were used as needed (i.e., semi-scripted). The timing of the data collection in this study also afforded a unique opportunity to discuss postdoc experiences related to the COVID-19 pandemic.

All sessions were conducted online and transcribed via Zoom [San Jose, CA]. Data from each participant group were compiled into a single corpus for that group (e.g., faculty advisors) prior to analysis. One member of the research team used inductive thematic coding to identify semantic themes within each set of focus group data [19, 20]. This type of thematic analysis provided a description of what existed in the data and allowed for the data to be organized into interpretative patterns [19]. As themes emerged through the analytic process, their connection to the identity-trajectory framework were noted, and thus subsequently mapped to the framework. This served as additional condensing of the data and grounded the findings in existing theory. Saturation of themes was achieved, providing a rich description of the entire data set, and themes were pervasive across participant groups [19–21]. A second reviewer independently audited a subset of the data using the codebook developed by the first reviewer. Agreement between the coder and auditor exceeded the established threshold of 80%; consensus building was used to resolve any discrepancies.

This study was submitted to the University of North Carolina Institutional Review Board (IRB) and determined to be not human subjects research (IRB #20–0817). Informed written consent was obtained prior to the start of the focus group or interview.

## Results

Postdocs specialized in clinical/industry (*n* = 12, 60%), bench/basic science (*n* = 4, 20%), and education/academia (*n* = 4, 20%). Eleven postdocs (55%) were in their first year of training and nine postdocs (45%) were in their second year of training or greater. Faculty advisers specialized in bench/basic (*n* = 4, 50%), clinical/industry (*n* = 3, 38.5%), and education/academia (*n* = 1, 12.5%). All faculty advisors (*n* = 8, 100%) had served as a mentor for more than one postdoc during their academic career.

Fourteen themes emerged from the data (Table 1). The themes mapped to all 4 constructs of the identity-trajectory framework. In addition, participants provided areas of opportunity and insight into experiences during the COVID-19 pandemic. Themes are described in more detail below and representative quotes are provided to further illustrate the findings.

**Table 1:** Findings from postdoc focus groups, mapped to the identity-trajectory framework [6, 11]

Element^a^	Definition^a^	Themes Identified	Example Quotes
***Intellectual***	Contributions to one’s disciplinary specialism	• Knowledge	*-Definitely gained a lot of new skills in different spaces—like how to write a grant and all the steps that are involved in doing that…* *-The biggest thing for me is just confidence and competence and my own autonomy and self-efficacy within academia*
		• Social and Behavioral Skills	
		• Technical Skills	
***Networking***	Work relationships and connections to the larger scholarly community	• Interdisciplinary Collaboration	*-Sometimes just asking your peers their experiences, you can just learn so much about what kind of research they're doing, their involvement, and [you’re] growing and building your own contacts and just getting to know people*
		• Networking with Alumni	
		• Postdoc Community	
***Institutional***	Fulfilling institutional responsibilities and accessing resources	• Mentor/Supervisor Support	*- I have been a part of the UNC postdoc association. There are some things that as an incoming international postdoc people have to understand on their own—for example, taxes* *- All of my mentors have put me in front of opportunities to progress me in my career*
		• Program Expectations	
		• School Support	
		• University Support	
***Agency***	Motivations, intentions and efforts to move forward given structural or systematic factors beyond their control	• Career Plans/Trajectory	*-When you can see the direct relation between what you're doing as a trainee and how this can help you in the future for your professional career, I think that's been a huge motivator and it definitely contributes to [my] happiness*
		• Postdoc Goals	
*Agency—Institutional*	Personal strategies for recognizing, navigating, or leveraging institutional aspects of the program	• Program Fit	*-And part of whether or not you survive in that environment is the support of your mentor and their guidance and helping you figure out: Oh, I've stepped in a big mud puddle here; I didn't know that was important to this person; or Oh, I should raise my hand and speak up here and offer insight on this*
		• Transitions (e.g., to and from postdoc training)	

^a^Adapted from Chen, McAlpine, and Amundsen, 2015 [11]

### Intellectual

Three themes emerged related to the intellectual strand: knowledge; technical skills; and social and behavioral (also called “soft”) skills. Technical skills included various research methodologies, software, and bench techniques. Social and behavioral skills, such as collaboration, communication, confidence, and autonomy were discussed. Participants widely acknowledged the intellectual strengths of their training programs and described opportunities to acquire the knowledge and skills needed for specialized expertise. Faculty participants specifically acknowledged the critical intellectual role (e.g., knowledge, technical skills) that postdocs play in supporting faculty and graduate students. While the intellectual themes were often described independently of one another, participants acknowledged the importance of all three. As noted by one postdoc, *I've been able to develop a lot of different soft skills, especially communication, teamwork, and time management related to the projects that I have in the research lab…I think it's a balance between hard skills and soft skills in this way.*

Of note, faculty and postdoc participants described the intellectual complexities associated with the various foci of the postdocs and postdoc training programs at the School (e.g., one faculty member stated that *the School has some unique training programs that attract postdocs…*). The diverse nature of postdoc backgrounds (e.g., terminal degrees, undergraduate and graduate experiences), programs, and research foci can present distinct challenges associated with meeting the various intellectual needs and interests of trainees. This was noted by one postdoc who commented that *the concerns are very different [based on research focus], the needs are very different, and the challenges I think are sometimes very different.* Due to these differences, some participants expressed interest in additional learning opportunities (e.g., coursework, workshops, symposia, teaching experiences) and professional development opportunities that would support their intellectual needs, diversify their skillsets, and uniquely position them for their career goals.

### Networking

Three themes were identified related to the networking strand: postdoc community; interdisciplinary collaboration; and networking with alumni. While some noted positive aspects of these themes within their training, others described these as areas of opportunity. As one postdoc shared, *I love that there’s a community of postdocs…at the School there’s so many different types of research that are ongoing but just not as many opportunities to get to know people*. Participants also acknowledged that networking needs and interests varied across the wide range of research foci at the School.

Faculty participants also identified networking as an area of opportunity yet focused primarily on postdoc community and networking with alumni themes. When speaking about the overall School community, one faculty participant acknowledged that *the postdocs kind of get left out a little bit*. In discussing what the School can do to better support advisors for postdocs, a faculty participant suggested *creating a network for postdocs to network with alumni…having more exposure to how different departments [in the School] work with postdocs…have events that help facilitate a sense of community/family*.

### Institutional

Four themes emerged related to the institutional strand: university support; school support; program expectations; and mentor/supervisor support. As it related to accessing resources, postdocs noted various university units available to support their training and development (e.g., Office of Postdoctoral Affairs, Odum Institute) and at the School (e.g., Center for Innovative Pharmacy Education and Research, Office of Organizational Diversity and Inclusion). However, faculty acknowledged that additional support and School infrastructure for postdocs could be useful. Postdocs advocated for a more cohesive and active postdoc community (e.g., synergy across various postdoc training programs) and improved postdoc communication across the School. Other recommendations included creating a School webpage dedicated to postdoc information and resources.

Postdocs and faculty believed that program expectations were generally clear. Although there were occasions when their experience (e.g., job duties, activities) did not align with anticipated expectations, postdocs described various ways in which their training afforded them flexibility and autonomy, with some positions evolving as needed over the course of the postdoc program. Overall, postdocs acknowledged feeling strongly supported by their advisors/supervisors, as noted by one postdoc, *I had a really fantastic mentor and he really helped me get a really well-rounded experience. So, I was involved in all sorts of activities like writing publications, doing presentations outside of T32 required presentations, mentoring students, helping him with his course development and instruction.*

### Agency

Four agency themes were identified: postdoc goals; career plans/trajectory; transitions; and program fit. Goals varied widely, including interests associated with presenting at conferences, submitting proposals for grants or postdoc awards, and teaching in courses. Postdocs acknowledged the role of the university, school, and mentor reputation as important motivating factors for joining the postdoc program, as reputation was seen as a mechanism for helping postdocs achieve their goals and career plans. Postdocs also confirmed their interest in various career foci and generally indicated that postdocs were being trained for common career trajectories (e.g., industry, academia).

Faculty advisors also discussed the importance of helping postdocs achieve their goals and career plans. As one faculty participant shared, *We use an IDP [Individual Development Plan]…to help have a better idea of what exactly [the postdoc’s] goals are, what they feel they’re doing well and not so well and we reflect on that at least once a year.* Another faculty mentor recommended having faculty and postdocs independently set postdoc goals prior to having *conversations about how [to] put those two things together*. While the specific goals of the postdoc training differed between the different types of postdocs, the faculty unified around goals such as understanding research design, gaining experience in scientific writing, understanding how to communicate findings, and being able to work independently as well as in a team.

Two themes were considered intersectional between agency and institutional, representing personal strategies for recognizing, navigating, or leveraging institutional aspects of the program. Transitions were described by postdocs as key points of development, presenting various challenges and opportunities that enabled postdocs to reflect and commit to their career goals. Transitions could include movement between any phase of education and career, including student to postdoc, residency to postdoc, and postdoc to career. Along the same lines, participants emphasized the importance of program fit, describing their programs as a unique experience that they could not get anywhere else and that would differentiate them for their career path.

### COVID-19 pandemic

COVID-19 themes aligned with the institutional and agency constructs of the identity-trajectory framework. Namely, institutional themes included university support, school support, and mentor/supervisor support. Postdoc participants lamented on the loss of School and social interactions while faculty expressed understanding in this disappointment. As one faculty participant shared, they made *sure to maintain connection throughout COVID.* Agency themes included transitions (specifically related to the transition to a remote work model) and postdoc goals (particularly as it relates to COVID-19 impact on motivation, well-being, and autonomy). In general, pandemic postdoc experiences varied widely depending on research specialization and often mirrored broader experiences of academia, cloaked in uncertainty and concern.

## Discussion

Postdocs play a critical role in the success and economic well-being of academic institutions [1–3]. Accordingly, the National Postdoctoral Association (NPA) was established in 2002 to give voice to postdocs and improve their training experiences [22]. By engaging various stakeholders, our work was able to explore the strengths, opportunities, and challenges associated with postdoc experiences and development within a research-intensive school of pharmacy. Namely, participants highlighted the intellectual merits of their training, networking opportunities, and institutional resources, clearly aligning the experiences of postdocs with the identity-trajectory framework. While these findings align with previous research about postdoc training, they also provide insight into specific challenges and opportunities. This study is the first known qualitative evaluation of postdoc experiences within pharmacy and pharmaceutical sciences education and contributes to a small corpus of literature regarding postdoc training in the health sciences.

Of note, the findings of this work align with studies that highlight the importance of career development and mentoring in postdoc training. For example, postdocs described their training programs as unique experiences that would differentiate them for their career path and they indicated an interest in additional learning opportunities that would position them for their career goals. A growing body of research elucidates the shifting job market and evolving career interests of postdocs, with fewer than 20% of postdocs entering permanent academic positions [1, 23]. Unfortunately, postdocs often feel confusion, uncertainty, and lack of support for their careers [24–26]. As research career interests extend beyond traditional academic roles – a reality reinforced by participants based on our findings – postdoc programs must align their strategies with these increasingly diverse career trajectories. While universities frequently offer career and professional development services for postdocs, they often lack systematic planning and implementation [23, 27] and may not be aligned with the unique needs of pharmacy postdocs.

This appeared true in our study, with postdocs and faculty mentors acknowledging the need for additional school-based support and infrastructure for postdocs. Given the critical role of mentors in postdoc development and success [12], efforts may be needed to ensure mentors are well-equipped to support postdocs for successful career advancement. In a study of minoritized postdocs, for example, researchers found that postdocs appreciated conversations about short- and long-term career goals with mentors and found more value in interactions with faculty who were flexible and open to innovative ideas [28].

Similarly, the findings of this study align with research that emphasizes the value of networking as an important part of postdoc success. Our participants identified networking as an area of opportunity, indicating a desire to engage more with peers, colleagues, and alumni. Åkerlind advocated for the development of supportive networks to counter the common postdoc experience of working in isolation [29]. While networking may include alumni and interprofessional colleagues, Chen et al. and Baiduc et al. also highlighted the importance of peer networking, which can improve postdoc satisfaction, enable smoother transitions, and enhance professional development. [11, 30] These sentiments align with recent literature on the impact of peer mentoring/support groups within pharmacy. Peer mentoring/support groups can provide a supportive environment that enhances participants professionally (e.g., increased writing productivity, helped navigate promotion expectations, provided a sounding board to resolve conflict among colleagues) and personally (e.g., provided support with work/life integration, helped decompress stress) [31, 32]. At the School, efforts are ongoing to promote community building and networking among postdocs, including regular social gatherings (e.g., bagels/coffee), cross-discipline seminars (e.g., mindfulness, self-compassion, resiliency, meditation) and guest panels (e.g., hybrid academic/industry/government). To build an even greater sense of community, most efforts have also been expanded to include graduate students.

Results that highlight the wide range and potential impact of postdoc backgrounds, contexts, and research foci in pharmacy and pharmaceutical sciences education are particularly noteworthy. Postdoc positions within health sciences schools in the United States are typically intellectually diverse, representing various disciplines with divergent research experience, exposure to formalized training, and methodological skills. This can differ from European training models, for example, in which completing a PhD, engaging in years of research, and publishing numerous studies are common prerequisites for postdoc positions. Postdocs have previously described the complexity of training based on differences in institution size, mandates, and disciplines [1]. Chen and colleagues, for example, found that agency was exercised differently by postdocs depending on desired careers and available resources [11]. Although comparisons between groups based on background (e.g., prior degree or research experience) or location (e.g., United States, Europe) was beyond the scope of our study, this could be an important next step for understanding postdoc experiences, and how to best support and foster their agency.

While postdoc experiences in this study were largely positive, we would be remiss to ignore known experiences that can negatively influence postdoc agency, including one’s wellbeing and feeling of inclusiveness [26, 33, 34]. During postdoc training, these feelings have been shown to manifest as imposter syndrome as well as a lack of connectedness or feeling unwelcome, particularly in those who identify with a minoritized racial or ethnic identity [26]. Experiencing these feelings can affect how postdocs perform during their training and prepare for the workforce. To help mitigate the feeling of imposter syndrome, findings from programs outside of pharmacy suggest designing professional development programs and individual development plans that focus on strengthening skills (e.g., academic writing). Since environmental factors can also promote imposter syndrome, the intentional creation of peer support groups and a sense of community may help those who experience this phenomenon [31–33]. This may be particularly important as postdocs experience transitions, which can be laborious and emotional in educational settings [35]. Mentor training on promoting inclusivity may also help ensure a positive postdoc experience for those who identify with a minority racial or ethnic identity. While none of the participants in this study explicitly expressed concerns regarding imposter syndrome or an unwelcoming environment, all programs should recognize that those issues are present or possible.

The postdoc identity-trajectory framework proved a useful fit for organizing and interpreting the data collected in this study [11]. This framework draws from the belief that the dynamic nature of individuals’ histories, expectations, and experiences influence their decision to invest in academic work. This aspect of the framework is critical for health sciences given the divergent backgrounds, interests, and experiences of postdocs mentioned above, highlighting the wide range of lived experiences among postdocs within a single school. However, the framework also juxtaposes individual agency against organizational structures that can introduce processes, requirements, and experiences beyond the individuals’ control [6]. The results of our study confirmed the reality of the tensions between individual agency and institutional support (e.g., valuing autonomy while simultaneously requesting additional support). Further, all themes identified in our analysis aligned with at least one of the four constructs of the framework, indicating that identity-trajectory appears well-suited for understanding postdoc training in pharmacy. It is worth noting, however, that there are other frameworks addressing various aspects of postdoc training. Ranieri and colleagues, for example, identified six factors that influence career progression in clinical postdocs: intrinsic motivation; work-life balance; inclusiveness; work environment; mentorship; and availability of funding [13]. In engineering, Mendez and colleagues found four elements of effective postdoctoral socialization: nurturing academic identity; reinforcing disciplinary belonging; strengthening scholarly performance; and providing career development for pursuing the professoriate [12]. Nowell and colleagues explicated intellectual and networking more deeply in the Professional Learning and Development framework for postdocs, which includes: professional socialization; professional skills; academic development; and personal effectiveness [1]. In addition, the NPA advocates for postdoctoral scholars to master competencies in discipline-specific conceptual knowledge, research skills, communication skills, professionalism, leadership and management skills and responsible conduct of research [2]. While the identity-trajectory framework aligned with our study, other frameworks may be useful for scholars and educators working to advance the development and evaluation of postdoctoral training.

The annual ‘Best Places to Work for Postdocs’ survey consistently shows that postdocs prefer institutions that listen and respond to postdoc concerns [4]. Program evaluations, including the use of qualitative data, are critical for ensuring that institutions both understand postdoc experiences and align their training programs with the needs of trainees, mentors, and schools [36]. Conducting focus groups with various stakeholders across the School enabled us to glean insight into postdoc training from various viewpoints, provided rich information about opportunities for strengthening training programs, and equipped School leadership to make data-informed improvements. Shortly after this evaluation, coupled with additional data collected independently, School leadership appointed a Director of Postdoctoral Programs to develop an infrastructure that more fully supports our postdocs. This is an administrative appointment for a current faculty member who is responsible for the engagement, connection, and development of the School’s postdoc community, developing new postdoc programs, overseeing programmatic engagement of postdocs within their programs, helping postdocs understand related policies and procedures, promoting the use of IDPs within postdoc training, overseeing the development and execution of strategic initiatives, programming, and policies aimed at improving postdoc engagement, and providing financial oversight of the postdoc programs.

This study has several limitations. First, this evaluation was conducted at a single, research-intensive institution with a relatively small sample size drawn from an intellectually diverse postdoc population. While this design limits generalizability, and the data may reflect local context and culture, the needs and experiences of these participants are likely reflected at similar types of institutions. Expanding this work to include additional institutions could be an important next step for further understanding postdoc experiences. Second, the participants interviewed were volunteers, which may have introduced self-selection bias. However, the qualitative design of this study enabled us to reach saturation among participants as the themes discussed appeared across all participant groups [19]. Third, the majority of postdocs and faculty at the School are White, limiting our understanding of how postdoc training might be experienced by individuals from marginalized groups. Fourth, social desirability bias could have occurred despite de-identifying data and ensuring anonymity in the dissemination of findings.

## Conclusion

This work contributes to a clear gap in the literature concerning postdoc experiences in pharmacy. The results of this work informed changes within the School and are offered in hopes of helping others support their postdoc programs. Postdocs and mentors could use these results to discuss professional learning plans and foster critical reflection on intellectual opportunities, networking needs, institutional resources, and postdoc agency. In addition, Schools could use these findings to guide program development and enhance institutional infrastructure for postdocs. Given their importance in pharmacy and pharmaceutical sciences, more focus on understanding and optimizing postdoc programs across the Academy is needed.

## Supplementary Information

**Additional file 1: Appendix 1.**

## Data Availability

The datasets generated and analyzed during the current study are not publicly available due to the small sample size and possibility of compromising anonymity/individual privacy; however, data may be made available from the corresponding author on reasonable request.
