# Supplementary material for: Qualitative evaluation of postdoctoral trainee and faculty advisor experiences within a research-intensive school of pharmacy
Source: BMC Med Educ. 2022 Sep 24;22:691. doi: 10.1186/s12909-022-03750-8 (PMC9509594; doi:10.1186/s12909-022-03750-8)
Supplement: Supplementary file 1 — Additional file 1: Appendix 1. [file 12909_2022_3750_MOESM1_ESM.docx]

**Script for Focus Groups (Postdocs):**

1. Main question: What attracted you to your current postdoc position?
   1. Probe for: alignment with expectations
2. Main question: What have you gained from your postdoc to date?
   1. Probe for: support for career readiness
   2. Probe for: support for job satisfaction/happiness
3. Main question: In your experience, what do you think is lacking in your postdoc?
4. Main question: What areas for improvement do you see?
   1. Probe for: School support (e.g., What can the school do to help better support postdocs?)
   2. Probe for: Programming (e.g., What types of programming for postdocs might be helpful?)
5. Main question: What has your experience been like as a postdoc during the COVID-19 pandemic?
   1. In what ways have you felt supported during this time? (by the PI, School, or University)
   2. In what ways have you not felt supported during this time?
6. Those are all of the questions we have for you. Is there anything else you would like to add?

Additional Probing Questions (use spontaneously when needed):

1. Can you tell me more about that?
2. Would you explain that further?
3. Can you give me an example?
4. Is there anything else?

**Script for Focus Groups (Faculty):**

1. Main question: Why do you have postdoctoral positions in your lab? What purpose do postdocs serve on your team?
2. Main question: What do you believe attracts postdocs to the UNC Eshelman School of Pharmacy?
3. Main question: What types of experiences do you believe contribute to your postdocs’ preparedness for their career?
   1. Probe for: supervisor goals (e.g., What do you hope are the main takeaways your postdocs leave with in professional skills? In research skills?)
   2. Probe for: postdoc goals (e.g., What do you think influences your postdoc happiness/satisfaction in the position?)
   3. Probe for: progression (e.g., How do you determine if your postdoc is progressing in their position?)
4. Main question: What strategies do you currently use to support your postdoctoral fellows?
   1. Probe for: school strategies (e.g., what does the School currently do that is beneficial? What structures are in place that are beneficial?
   2. Probe for: supervisor strategies (e.g., What strategies do you use to support your postdoc to achieve their career goals? What strategies do you use to support your postdocs’ happiness/satisfaction?)
5. Main question: What can be done to better support you as a mentor for your postdocs?
   1. Probe for: programming, seminars, training, etc (e.g., what types of programming would you like to see?)
6. OPTIONAL MAIN QUESTION: What do you believe can enhance your postdocs experience during their time here?
   1. Probe for: What do you think the school can do help better support your postdoctoral fellow/research associate?
7. Those are all of the questions we have for you. Is there anything else you would like to add?

Additional Probing Questions (use spontaneously when needed):

1. Can you tell me more about that?
2. Would you explain that further?
3. Can you give me an example?
4. Is there anything else?
